# Lewis x-Carrying O-glycans are Candidate Modulators for Conceptus Attachment in Pigs

**DOI:** 10.1093/biolre/ioac204

**Published:** 2022-11-19

**Authors:** Kun Han, Yulu Yue, Weiwei Wang, Feiyu Wang, Wengang Chai, Shuhong Zhao, Mei Yu

**Affiliations:** 1 Key Lab of Agricultural Animal Genetics, Breeding and Reproduction of Ministry of Education, College of Animal Science and Technology, Huazhong Agricultural University, Wuhan 430070, China; 2 Glycosciences Laboratory, Faculty of Medicine, Imperial College London, London W12 0NN, UK

## Abstract

Successful attachment of conceptus to the uterine luminal epithelium (LE) is crucial for establishing a functional placenta in pigs. However, the underlying mechanisms are yet to be elucidated. The uterine LE-conceptus interface is enriched in various glycoconjugates essential to implantation. Using MALDI-MS profiling, we identified for the first time the O-glycan repertoire in pig endometrium during the conceptus attachment stage. The expression pattern of blood group A, O(H), Lewis x, y, a, b (Le^x^, Le^y^, Le^a^, and Le^b^), the sialylated and sulfated Le^x^ antigens in the uterine LE-conceptus interface was assessed using immunofluorescence assays. Notably, the Le^x^-carrying O-glycans exhibited a temporal-spatial expression pattern. They were absent in the endometrium on estrous cycle days but strongly and spatially presented in the conceptus and uterine LE to which the conceptus apposes during the early conceptus attachment stage. In addition, Le^x^-carrying O-glycans were co-localized with secreted phosphoprotein 1 (SPP1), a well-characterized factor that plays a role in promoting conceptus attachment through interacting with integrin αVβ3 and integrin αVβ6. Meanwhile, the immunoprecipitation assays revealed an interaction between the Le^x^-carrying O-glycans and SPP1, integrin αV, and integrin β6. Furthermore, we provided evidence that the β1,4-galactosyltransferase 1 (B4GALT1) gene is a potential regulator for Le^x^ antigen expression in the uterine LE-conceptus interface during the early conceptus attachment stage. In conclusion, our findings show that Le^x^-carrying O-glycans, presumably dependent on *B4GALT1* gene expression, might modulate conceptus attachment by interacting with the SPP1-integrin receptor complex in pigs.

## Graphical abstract

**Graphical Abstract:**
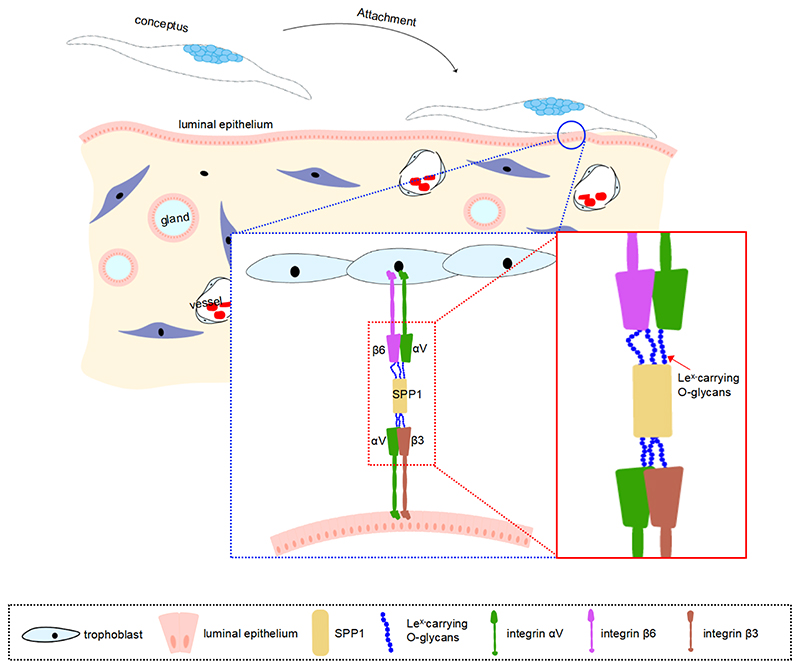

## Introduction

The implantation of an embryo to the endometrium is a critical step during the establishment of pregnancy. In pigs, maternal recognition is initiated by conceptus estrogens on Days 11 and 12, followed by adhesion of conceptuses to the uterine luminal epithelium (LE) until a stable attachment between conceptuses and uterine LE is established around Day 18 of pregnancy [1]. The important events during the sequential phases include apposition, adhesion, and forming a true epitheliochorial placenta [2]. Thus, a defect in the ability of the uterine LE to accommodate the conceptus adhesion is one of the major causes of implantation failure. Progress has been made to elucidate the mechanisms behind the transition of the uterine LE from an anti-adhesive state to an adhesive state [2–4]. Many adhesion molecules, such as secreted phosphoprotein 1 (SPP1, also called osteopontin, OPN), play essential roles in conceptus attachment [5, 6]. However, the mechanisms that lead to successful adhesion of the uterine LE by the conceptus in pigs remain to be fully elucidated.

The apical cell surface of the uterine LE and trophectoderm are heavily glycosylated. Glycan chains expressed at the uterine LE-trophectoderm interface are extensively involved in many implantation-related cellular processes, such as signal transduction and cell attachment [7–10]. In humans, the uterine LE-expressed sialyl Lewis x (SLe^x^) (detected with HECA-452 antibody) and 6-sulfo-SLe^x^ (detected with HECA-452 and MECA-79 antibodies) can be bound by L-selectin-bearing trophoblast to mediate the embryo adhesion [11], indicating that Lewis antigens have important implications in embryo implantation. Two glycan-binding proteins, galectin-15 and L-selectin, were expressed in sheep and bovine endometrium and proposed to play an embryo adhesion role through binding to their ligands on conceptus [12, 13]. In addition, glycosylation is also involved in gene transcription. For example, histone GlcNAcylation in H2B plays a role in transcriptional activation by promoting the H2BK120 monoubiquitination [14]. Therefore, characterization of the implantation-associated glycans and the genes regulating the formation of the glycan structures can provide important evidence for elucidation of the mechanisms involved in pig implantation.

N- and O-glycosylations are the two major post-translational modifications in eukaryotes. N-glycosylation is formed by the attachment of β-N-acetylglucosamine (GlcNAc) to a nitrogen atom in the asparagine residue, while O-glycosylation involves the extension of glycan chains to the oxygen of the hydroxyl group of serine or threonine residues. Because the sequences and diversity of the O-glycans are more complex, the study of O-glycosylation lags behind N-glycosylation [8, 15]. In pigs, many glycans were detected in the maternal-fetal interface using the lectin histochemistry approach [16, 17]. In addition, the O-glycan repertoires of several tissues and cells, including the heart (the pericardium, pulmonary and aortic valves), kidney, gastric tissues, and endothelial cells, have been identified [18–20]. Recently, the N-glycans in the pig endometrium from the implantation period were characterized by matrix-assisted laser desorption/ionization mass spectrometry (MALDI-MS) profiling [21]. However, the O-glycans in pig endometrium have not been well described. In this study, we generated the O-glycan repertoire of pig endometrium, investigated the association of O-glycan expression with conceptus attachment, and identified the genes determining the expression of O-glycan structures in pig uterine LE-conceptus interface during the conceptus attachment stage.

## Materials and Methods

### Ethics statement

The Ethics Committee of Huazhong Agricultural University approved all animal procedures (HZAUSW-2016-015).

### Sample Collection

The Yorkshire gilts used in the study were from Days 12 (CD12, n = 3) and 15 (CD15, n = 3) of the estrus cycle and Days 12 (GD12, n = 3) and 15 (GD15, n = 4) of pregnancy. The gilts were checked for estrus twice daily. They were bred at the onset of the second estrus (Day 0) and again 12 h later. The sample collection approach was described in our previous study [2], and a brief schematic diagram of sample collection is shown in Supplementary Figure 1. Briefly, the uterus taken from each pregnant gilt was quickly cut into 12-15 cm segments with sterile scalpel blades. Half of the uterine segments were randomly selected and flushed with cold RNase-free phosphate buffer saline (PBS). If the conceptuses were observed in uterine flushing, the uterus was opened longitudinally along the anti-mesometrial side. Then the endometrial tissue was collected from the middle region of the mesometrial side, snap-frozen in liquid nitrogen, and stored at -80°C. In addition, the remaining uterine segments, which were not flushed, were cut into pieces and either fixed with 10% neutral-buffered formalin (1 liter) in a specimen jar followed by paraffin embedding or embedded in precooled optimum cutting temperature OCT compound (SAKURA Tissue-Tek O.C.T. Compound 4583). The samples were then snap-frozen in liquid nitrogen and stored at -80°C. The intact uterus from Days 12 and 15 of the estrus cycle was cut into segments and fixed in 10% neutral-buffered formalin before embedding in paraffin. In addition, the small intestine tissues, which were used as a positive control of blood group A and O(H) antigens, were collected from the gilts on Day 15 of pregnancy and fixed in 10% neutral-buffered formalin before embedding in paraffin.

### Isolation of O-glycans from endometrial tissues

The frozen endometrial tissues from 3 gilts on Day 15 of pregnancy were pooled in equal weight. First, 500 mg of the pooled tissue sample was homogenized in denaturing buffer (0.5 M Tris-HCl, pH 8.5, containing 7 M guanidine-HCl and 10 mM EDTA) using an Ultrasonic Processor (Sonics VCX130, Sonics & Materials Inc, USA). Then the homogenized sample was centrifuged at 12,000 rpm for 35 min, and the supernatant was collected and lyophilized for further analysis. Second, 20 mg of the lyophilized sample was reduced with 10 mM DL-Dithiothreitol (DTT: protein (1:1 w/w)) at 60°C for 120 min, alkylated with 20 mM iodoacetamide (IAA: protein (2.5:1 w/w) at room temperature for 90 min in the dark, dialyzed against 20 mM ammonium bicarbonate (pH 8.6) for 48 h, digested with TPCK treated trypsin (T1426, Sigma, USA) at 37°C overnight to obtain glycopeptides. Third, to remove N-glycans, the glycopeptides were incubated with 3 mU peptide N-glycanase F (PNGase F, P0704S, New England Biolabs, MA, UK) at 37°C 16 h, and the O-glycopeptides were purified by Sep-Pak C18 cartridge (WAT054955, Waters, Ireland). Finally, the O-glycans were released by Carlson degradation (500 μL 0.05 M NaOH/1.0 M NaBH4 for 16 h at 45°C) [22]. The reducing reagent was destroyed by acetic acid, and the resulting borate was removed by repeated co-evaporation with methanol. The released O-glycan alditols was purified by a short cation exchange column and the eluant was lyophilized.

### Analysis of O-glycans using MALDI-mass spectrometry

Dried O-glycans (2 mg) were permethylated, as described previously [23]. Briefly, 150 μL DMSO/sodium hydroxide slurry was added to the dried O-glycan sample, and 50 μL methyl iodide was added to the mixture. The reaction mixture was vigorously shaken for 15 min at room temperature. The reaction was quenched by adding ultra-pure water. Then the mixture was extracted five times using chloroform. The pooled chloroform phase was dried under a gentle stream of nitrogen. The permethylated O-glycans were loaded onto a Sep-Pak C18 cartridge (WAT054955, Waters, Ireland), which was preconditioned sequentially with methanol, ultra-pure water, and acetonitrile, and eluted stepwise with water and 50% acetonitrile. The purified permethylated O-glycans were analyzed using a MALDI mass spectrometer with a QIT-TOF configuration (Shimadzu AXIMA Resonance, Japan) in the positive-ion mode. The O-Glycan annotation was made using GlycoWorkbench software [24].

### Immunofluorescence assays

The immunofluorescence assays were performed as described before [2, 25]. The paraffin-embedded uterine samples were cross-sectioned into 4 μm-thick slices using a Leica microtome (RM2235, Leica, Germany). The slices were deparaffinized in xylene, rehydrated through a series of increasing graded ethanol, submitted to heat-induced epitope retrieval by microwave treatment in 0.01 M sodium citrate buffer (pH 6.0), and rinsed three times in phosphate-buffered saline (PBS). Then the slices were blocked with 5% bovine serum albumin (BSA) for 30 min in a humid chamber, incubated with the primary antibody diluted in PBS overnight at 4°C, rinsed, and then incubated with the secondary antibody for 30 min. DAPI (G1012, Servicebio, Wuhan, China) was added for 10 min to visualize the nuclei. The primary antibodies used include (1) carbohydrate-specific antibodies to ABO(H) blood group and Lewis-related antigens. (2) protein-specific antibodies. Negative control (NC) was carried out by replacing the primary antibody with corresponding nonspecific immunoglobulin. Information on the primary and secondary antibodies and the negative controls used in the study was listed in Supplementary Table 1. All slides were scanned by a Pannoramic Midi slide scanner (3D HISTECH, Budapest, Hungary). The whole cross-sectional images were taken and visualized using the CaseViewer 2.0 software (3D HISTECH, Budapest, Hungary).

### Immunoprecipitation and western blotting assays

Each snap-frozen OCT-embedded uterine sample collected from Days 12 and 15 of pregnancy consisted of three components: the myometrium, endometrium, and conceptuses. The myometrium was removed at -30 °C. The rest of the sample, including the endometrium and the conceptuses wrapped by the endometrium (named intact endometrium-conceptus tissue sample), was used for immunoprecipitation and western blotting assays (Supplementary Figure 1).

Six intact endometrium-conceptus tissue samples were used for the immunoprecipitation assays (n = 3 gilts/day). Each of the samples (500 mg) was lysed in RIPA lysis buffer (P0013D, Beyotime, Shanghai, China) supplemented with 0.1% phenylmethylsulfonyl fluoride protease inhibitor (ST506, Beyotime, Shanghai, China) for 30 minutes. Then the lysate was centrifuged at 12,000 g for 30 min at 4C. The obtained supernatant (400 μL) was incubated with protein L magnetic beads (50 μL, 88849, Thermo Scientific, Waltham, USA) conjugated with anti-CD15 (recognizes Le^x^ antigen, ab665, Abcam, Cambridge, UK) on a rotator at 4°C overnight. The beads were washed five times with RIPA lysis buffer. The immunoprecipitated proteins were resolved by 10% SDS-PAGE, then transferred to the polyvinylidene difluoride (PVDF) membrane. The PVDF membrane was incubated with a primary antibody at 4°C overnight and a relevant second antibody at room temperature for 2 h. Information on the primary antibodies against SPP1, integrin αV, integrin β6, and integrin β3 was shown in Supplementary Table 1. The second antibody used was HRP-conjugated goat anti-rabbit IgG (Clean-Blot™ IP Detection Reagent, 1:500, 21230, Thermo Scientific, Waltham, USA). In addition, the expression of the proteins in the endometrium-conceptus tissue samples was determined by western blotting assays. β-actin (1:2500, AF5003, Beyotime, Shanghai, China) was used as the loading control. The proteins were detected using the ECL Western blotting kit (170-5060, Bio-red, California, USA) and visualized by a chemiluminescent imaging system (Tanon-5200, Tanon Science and Technology, Shanghai, China).

### Bioinformatic analysis

A recent study reported the RNA-seq data of the pig uterine LE from the mesometrial side (M) and the anti-mesometrial side (AM) on Days 12 and 15 of pregnancy (PRJNA668716) [2]. In this study, the glycosyltransferase genes with FPKM (Fragments per kilobase of transcript per million mapped fragments) greater than 10 were retrieved from the RNA-seq data.

The ChIP-seq bigwig output files for H3K27ac and H3K4me3 of eight pig tissues (heart, kidney, liver, lung, muscle, pancreas, spleen, and thymus) were downloaded from NCBI Gene Expression Omnibus (GEO) database (GSE143288) [26]. The bigwig files were analyzed using the Integrative Genomics Viewer (IGV) to visualize the enrichment regions of H3K27ac and H3K4me3 in *B4GALT1* gene (SSC10: 33,317,295-33,381,285; *Sus scrofa* genome assembly 11.1).

### Chromatin Immunoprecipitation (ChIP) assays

The ChIP assays were performed on the intact endometrium-conceptus tissue samples, the same as those used for the immunoprecipitation and western blotting assays (n = 3 gilts/day). Each sample (250 mg) was fixed with 1% formaldehyde for 10 min at room temperature. The reaction was quenched by adding glycine (final concentration 0.125 M), washed with ice-cold PBS, and lysed in lysis buffer (50 mM HEPES, 150 mM NaCl, 1 mM EDTA, and 1% TritonX-100, 0.1% SOD, and 1% SDS) to obtain chromatin. The chromatin was sonicated using Ultrasonic Processor (Sonics VCX130) and incubated with antibodies against H3K27ac or H3K4me3 conjugated magnetic beads on a rotator at 4°C overnight. Information on the antibodies was shown in Supplementary Table 1. The beads were washed and de-crosslinked by resuspending the beads in a buffer containing 200 μL of 1% SDS, 1 M EDTA, and 10 mM Tris-HCl (pH 8.0) at 65°C for 30 min. The supernatant was incubated with 10 μL proteinase K at 55°C for 6 h. Non-immunoprecipitated chromatin was used as the total input control. The ChIP-DNA and non-immunoprecipitated control (input) were purified by DNA Extraction Reagent (P1012, Solarbio, China) for further ChIP-qPCR analysis.

### Quantitative Real-Time PCR (qRT-PCR) and ChIP-qPCR

The qRT-PCR was performed to detect the expression of *B4GALT1* gene in the intact endometrium-conceptus tissue samples (Supplementary Figure 1). First, total RNA was isolated using the RNeasy Plus Mini Kit (Qiagen, Germany) (n = 3 gilts/day), then the cDNA was synthesized using PrimeScript RT Reagent Kit with gDNA Eraser (RR047B; Takara Biomedical Technology, Shiga, Japan). The *GAPDH* gene was used as a control. In addition, to validate the enrichment regions of H3K27ac and H3K4me3 in *B4GALT1* gene, DNAs from either the immunoprecipitated chromatin or non-immunoprecipitated chromatin (input) were subjected to the ChIP-qPCR analysis.

The qRT-PCR and ChIP-qPCR were performed using SYBR Premix Ex Taq (Takara Biomedical Technology, Shiga, Japan) in a Bio-Rad CFX96 Touch Real-Time PCR Detection System (Bio-Rad Laboratories, Inc., Hercules, CA, USA). The primers used for qRT-PCR and ChIP-qPCR were provided in Supplementary Table 2. The comparisons between Days of pregnancy were carried out using Student’s *t*-test. Differences were considered to be significant if *P* < 0.05. Statistical analyses were performed with GraphPad Prism Software version 5 (GraphPad Software, San Diego, CA, USA).

## Results

### The O-glycans expressed in pig endometrium during the attachment stage

**Figure 1:**
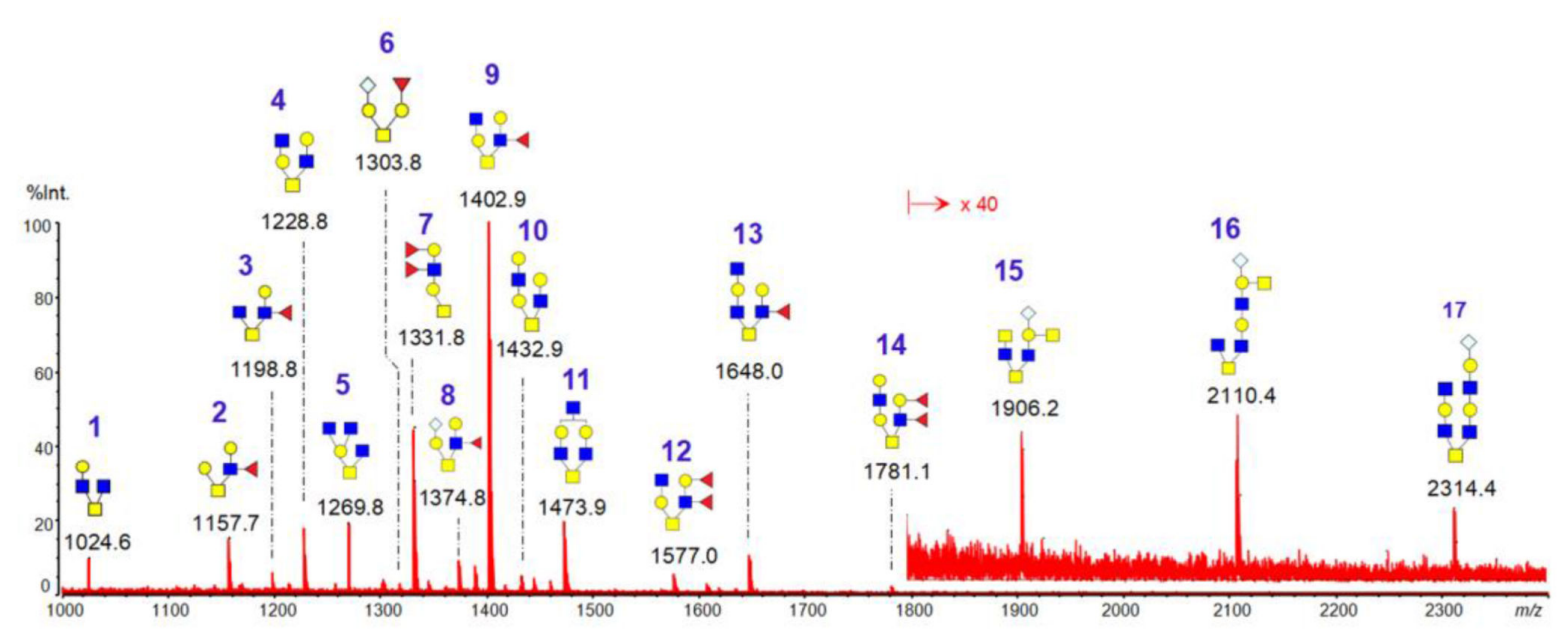
MALDI mass spectrum of permethylated O-glycans isolated from pig endometrium on Day 15 of pregnancy. Each ion peak, [M+Na]^+^, represents an O-glycan composition. The peak number is blue, and the m/z value is black. The mass range of 1800 to 2300 is magnified 40 times.

The O-glycans in pig endometrium on Day 15 of pregnancy were characterized by MALDI-MS profiling. As shown in Figure 1 and Table 1, the O-glycans range in size from tetra- to nonasacccharides. Among the identified 17 O-glycan species, five (peaks #6, #8, #15, #16, and #17) contain terminal N-glycolylneuraminic acid (Neu5Gc), and eight have one (#2, #3, #6, #9, and #13) or two (#7, #12, and #14) terminal fucose (Fuc) residues. Thus, the O-glycans in pig endometrium during the attachment stage are primarily fucosylated.

### Expression of fucose-containing O-glycans in pig uterine LE-conceptus interface

The natures of the terminal fucose-containing epitopes in O-glycans in pig uterine LE-conceptus interface were examined using immunofluorescence assays with antibodies specific for AO(H) blood group or Lewis antigens (Supplementary Table S1). As pigs are known to have blood group A and O(H) antigens [27], the anti-A and H antibodies were initially used. As expected, positive staining of A and H antigens was detected in the intestinal tissue (Supplementary Figure 2A), validating the immunostaining method used in the present study [28]. Subsequently, we found that the H antigen was not expressed in uterine LE but weakly expressed in the glandular epithelium (Supplementary Figure 2B). However, two different expression patterns of the A antigen were detected among pregnant gilts on Days 12 and 15 investigated: (1) on Day 12, the A antigen was expressed in uterine LE and glandular LE but absent in the vessels; (2) on Day 15, the A antigen was absent in uterine LE and glandular LE but present in the vessels (Supplementary Figure 2C). Taken together, the blood group O(H) antigen was not expressed, but the A antigen showed an inter-individual variation in expression in pig uterine LE on Days 12 and 15 of pregnancy.

**Figure 2:**
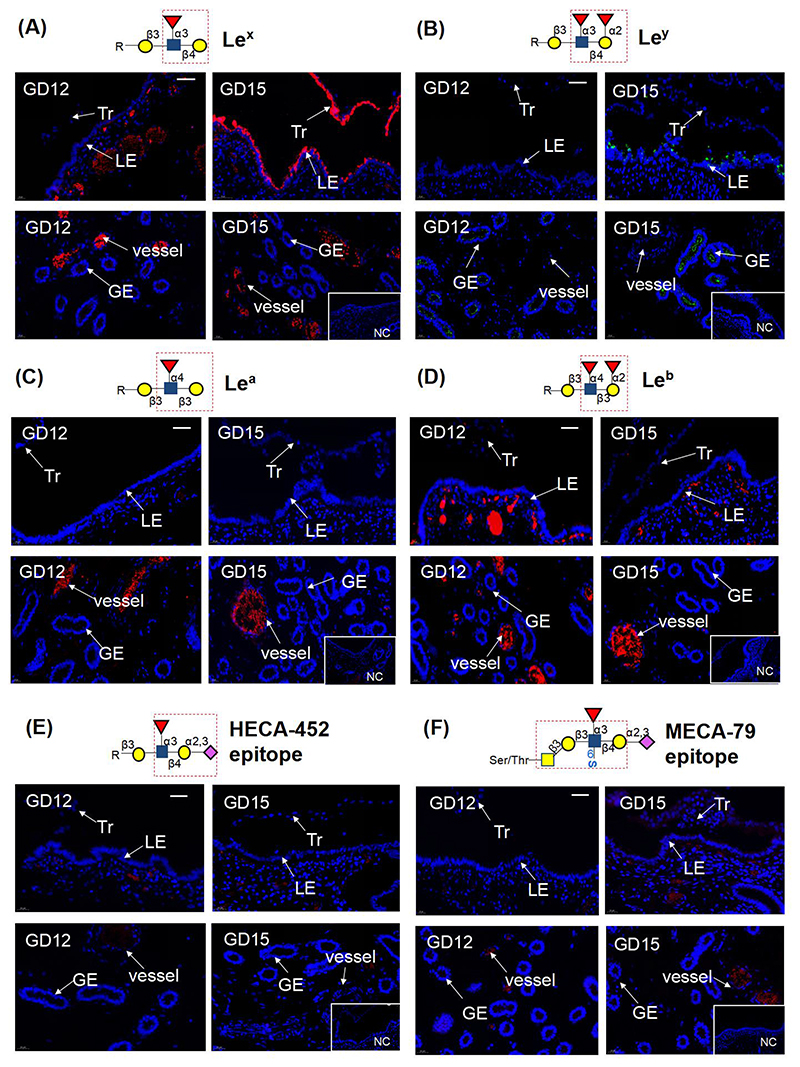
Representative images show the expression of Lewis-related antigens in uterine LE and conceptus (top row of each panel) and other compartments of the endometrium (bottom row of each panel) in the uterine cross-sections from Days 12 and 15 of pregnancy in pigs (n = 3-4 gilts/Day of pregnancy). The schematic diagram showing the structure of each antigen detected in this study is placed on the top of each panel. NC, negative control. (A) Le^x^ antigen. (B) Le^y^ antigen. (C) Le^a^ antigen. (D) Le^b^ antigen. (E) HECA-452 epitope. (F) MECA-79 epitope. Fucose 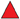. N-acetylglucosamine 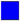. N-acetylgalactosamine 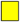. Galactose 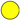. sialic acid 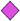. S, sulphate. LE, luminal epithelium. GE, glandular luminal epithelium. Tr, trophoblast. GD, Day of pregnancy. Scale bar = 50 μm. The antibodies and the negative controls are listed in Supplementary Table 1.

We further looked at the possible expression of Lewis antigens (Le^x^, Le^y^, Le^a^, Le^b^, sialyl-Le^x^, and 6-sulfo-SLe^x^). We found that the expression of Le^x^ antigen was undetectable in the uterine LE on day 12 but abundantly expressed in pig uterine LE and the conceptus on Day 15 (Figure 2A). It must be noted that the Le^x^ could attach to either the N- or O-glycans. As the non-reducing terminal α1-3- and α1-2-fucosylated N-glycans are absent in pig endometrium during implantation [21], it can be concluded that the Le^x^ antigen we detected is on O-glycans in pig uterine LE and the conceptus on Day 15. In addition, Le^y^ was expressed very weakly in the uterine LE of pregnant gilts on Days 12 and 15 but strongly in the glandular epithelium on Day 15 (Figure 2B). However, the other two Lewis antigens (Le^a^ and Le^b^), the SLe^x^ (detected with HECA-452 antibody) and 6-sulfo-SLe^x^ (detected with HECA-452 and MECA-79 antibodies), were utterly absent in pig uterine LE and other endometrial compartments (Figures 2C, 2D, 2E, and 2F).

### Association of Lewis x-carrying O-glycans and the conceptus attachment

**Figure 3:**
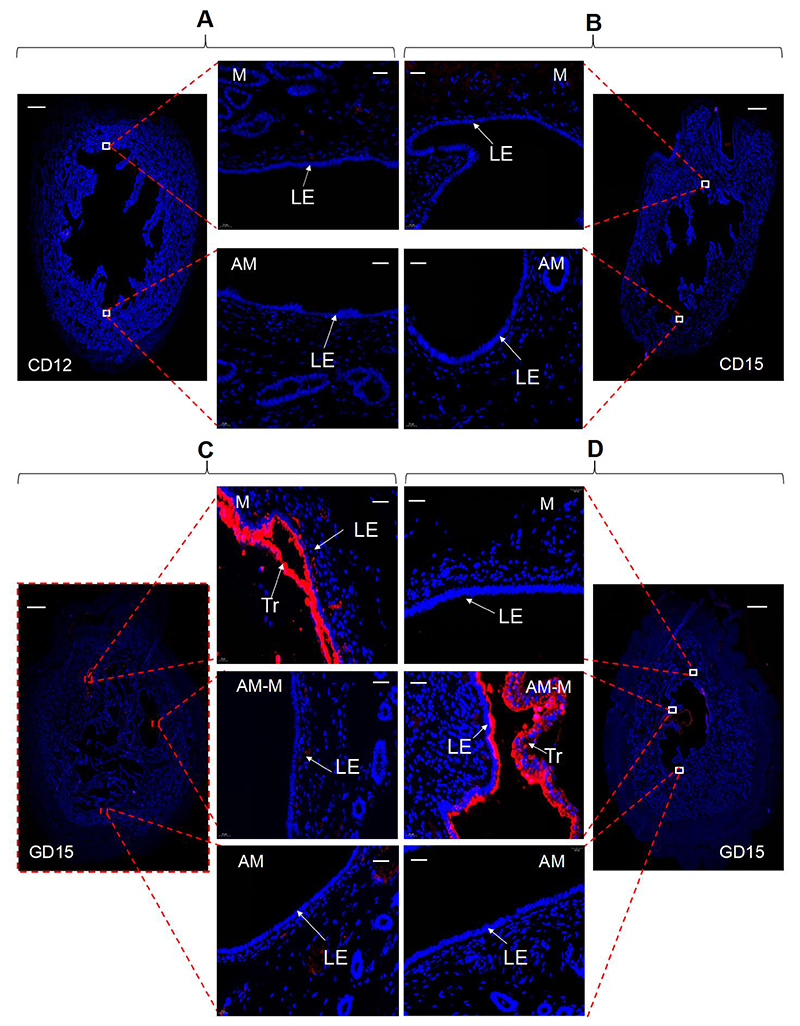
Expression of Le^x^-carrying O-glycans in the uterine cross-sections from estrous cycle Day 12 (A) and estrous cycle Day 15 (B), or in the uterine cross-sections from Day 15 of pregnancy in which the conceptus attaches to the mesometrial side (C) or a site away from the mesometrial side (D). White squares in each stained uterine cross-section (scale bar = 2000 μm) specify the areas shown at higher magnification (scale bar = 50 μm). n = 3-4 gilts/ estrous cycle Day or type of uterine cross-sections defined according to where the conceptus is located within the uterus. M, mesometrial side. AM-M, between the mesometrial and anti-mesometrial side. AM, anti-mesometrial side. LE, luminal epithelium. Tr, trophoblast. The antibodies and the negative controls used are listed in Supplementary Table 1.

We further investigated whether Le^x^ in O-glycans was associated with the conceptus attachment because it was intensely present in pig uterine LE and conceptus on Day 15. First, we found that Le^x^ was not expressed in pig uterine cross-sections on estrous cycle Days 12 and 15 (Figures 3A and 3B). Subsequently, we examined the expression of Le^x^ in the two types of uterine cross-sections defined according to where the conceptus is located within the uterus. As shown in Figures 3C and 3D, wherever the conceptus attaches on Day 15 of pregnancy, Le^x^ was always expressed in the apical surface of the uterine LE-conceptus interface but absent in the uterine LE without adjacent conceptus.

### Expression of the selectins in the uterine LE-conceptus interface

Fucosylated glycans can facilitate ligand-receptor interactions to mediate cell-cell adhesion. It has been shown that three members of the selectin family, P-selectin, E-selectin, and L-selectin, could bind to fucosylated glycan structures [8]. Therefore, the immunofluorescence assays were performed to investigate their expressions on Days 12 and 15 of pregnancy. The results showed that all three selectins were not expressed in the uterine LE and conceptus (Supplementary Figure 3).

### Interactions of Lewis x-carrying O-glycans with SPP1-integrin receptors

**Figure 4:**
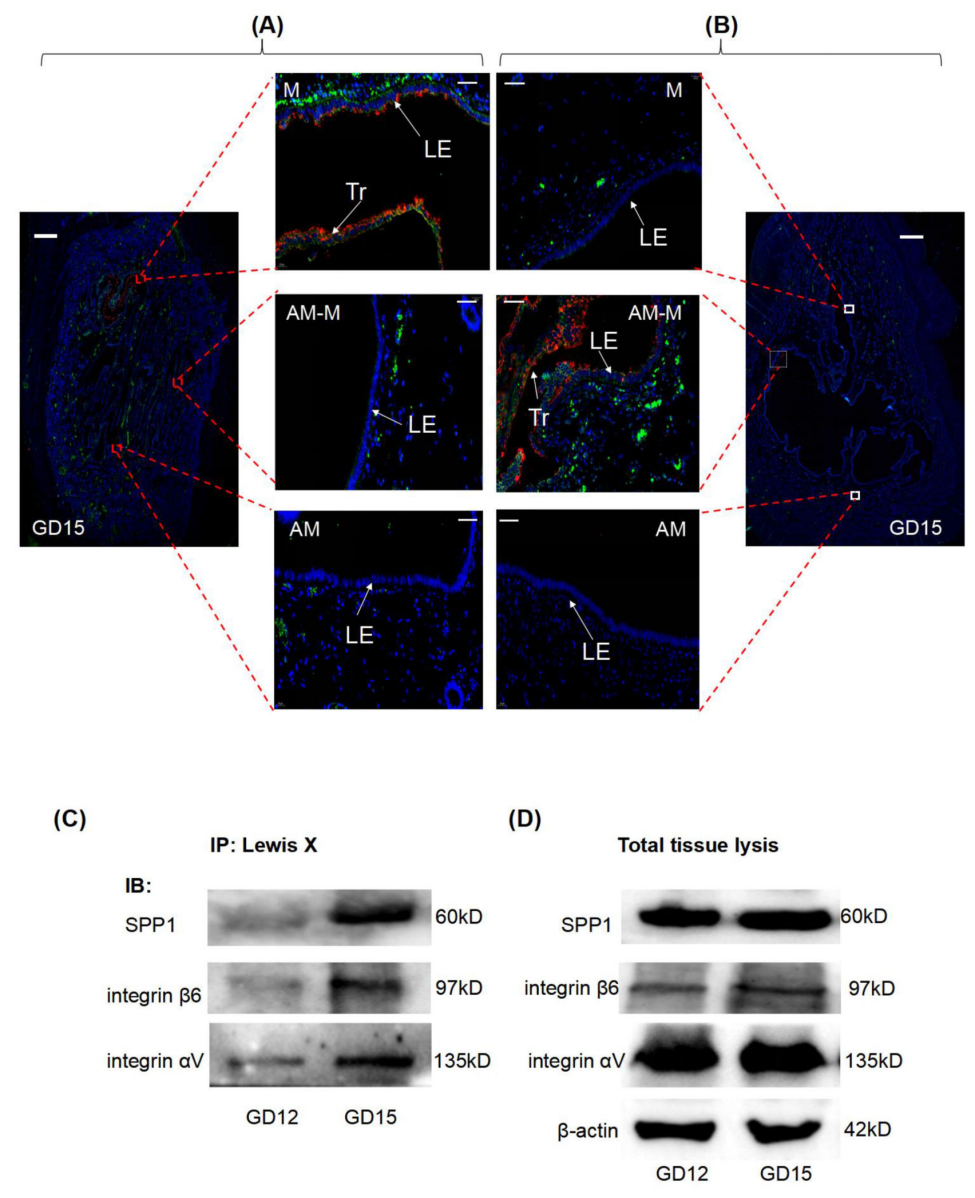
SPP1 and its integrin receptors interact with Le^x^-carrying O-glycans. Representative images show the co-localization of Le^x^-carrying O-glycans (red) and SPP1 (green) in the uterine LE-conceptus interface on Day 15 in which the conceptus attaches to the mesometrial side (A) or a site away from the mesometrial side (B). White squares in each stained uterine cross-section (scale bar = 2000 μm) specify the areas shown at higher magnification (scale bar = 50 μm). n = 3 gilts/ type of uterine cross-sections defined according to where the conceptus is located within the uterus. M, mesometrial side. AM-M, between the mesometrial and anti-mesometrial side. AM, anti-mesometrial side. LE, luminal epithelium. Tr, trophoblast. (C) Representative images show the immunoprecipitation results (n=3 gilts). The proteins interacting with Le^x^-carrying O-glycans in endometrium-conceptus tissue samples were pulled down with anti-CD15 and subjected to Western blotting. (D) Representative images show the amounts of the proteins. The tissue lysates were probed with anti-SPPl, anti-integrin αV, and anti-integrin β6. β-actin was used as a loading control. IP, immunoprecipitation. IB, immunoblot. The antibodies and the negative controls used are listed in Supplementary Table 1.

SPP1 and its receptors, uterine luminal epithelial integrin αVβ3 and trophectoderm integrin αVβ6, play essential roles in conceptus attachment in pigs [29]. We then investigated whether the Le^x^-carrying O-glycans interact with the SPP1-integrin receptor complex during the attachment stage. First, we observed that SPP1 and Le^x^ antigen were co-localized at the uterine LE-conceptus interface on Day 15 but were absent in the uterine LE on estrous cycle Day 15 (Figures 4A and 4B, Supplementary Figure 4). Next, the Le^x^-carrying glycoproteins were pulled down from the intact pig endometrium-conceptus tissue samples. The western blotting analysis showed that integrin β3 was not detected in the immunoprecipitates from either Day 15 or Day 12 (data not shown). However, SPP1, integrin αV, and integrin β6 were strongly present in the immunoprecipitates from Day 15 but weakly present in those from Day 12 of pregnancy (Figures 4C and 4D, Supplementary Figure 5).

### Regulators for Lewis x antigen expressions in pig uterine LE

**Figure 5:**
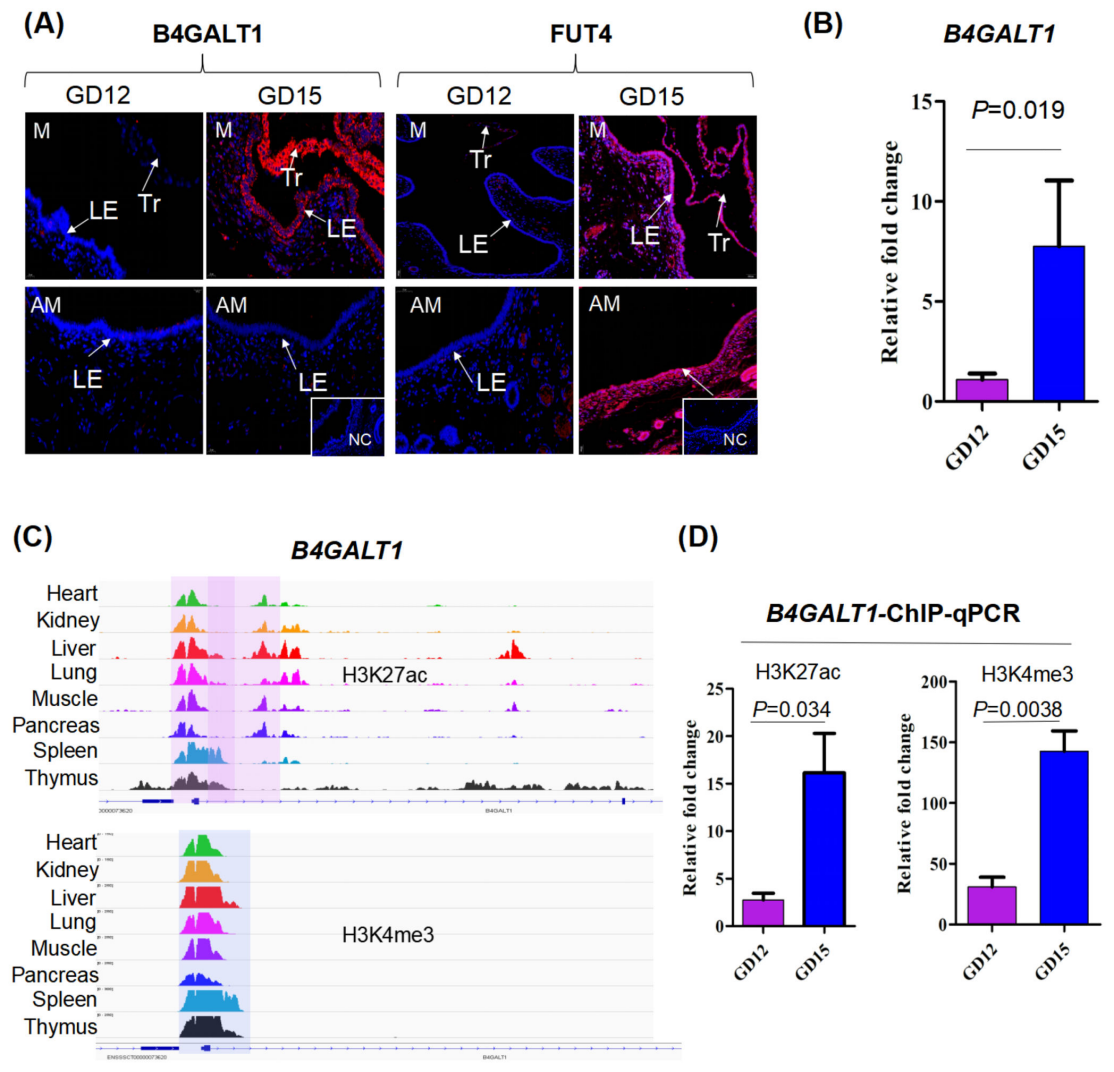
Analysis of the regulators for the expression of Le^x^-carrying O-glycans. (A) Representative images show the expression of B4GALT1 and FUT4 in uterine LE and conceptus at the mesometrial side (M; the top row of the panel) and anti-mesometrial side (AM; bottom row of the panel) in the uterine cross-sections from Days 12 and 15 of pregnancy in pigs (n = 3 gilts/Day of pregnancy). NC, negative control. The antibodies and the negative controls used are listed in Supplementary Table 1. (B) qRT-PCR analysis of the expression of the *B4GALT1* gene. (C) Genome browser views of H3K27ac and H3K4me3 modification regions in *B4GALT1* gene (*Sus scrofa* genome assembly 11.1). (D) ChIP-qPCR analysis of the enrichment of H3K27ac and H3K4me3 marks in *B4GALT1* gene. M, mesometrial side. AM, anti-mesometrial side. LE, luminal epithelium. Tr, trophoblast. GD, Day of pregnancy.

We next investigated the regulators that may determine the spatio-temporal expression pattern of Le^x^ antigen. Structurally, Le^x^ antigen (Galβ1-4(Fucα1-3)GlcNAcβ1-) is produced by transferring the α1,3-fucose residue to a structure that ends with a Galβ1-4GlcNAcβ1-R sequence. Beta-1,4-galactosyltransferases (B4GALTs) and fucosyltransferases (FUTs) participate in catalyzing the synthesis processes (Complex Carbohydrate Research Center database. Supplementary Figure 6A). First, we retrieved the two types of glycosyltransferase genes that were expressed in pig uterine LE from our previously published RNA-seq data [2]. The genes included *B4GALT1*, *B4GALT2*, and *FUT4* (Supplementary Figure 6B). Subsequently, immunofluorescence assays were performed to detect the expression of the three enzymes in the uterine cross-sections from pregnant gilts. Because positive stainings for B4GALT1 and FUT4 but not B4GALT2 were observed (Figure 5A, Supplementary Figure 6C), the expression patterns of B4GALT1 and FUT4 were further investigated. As shown in Figure 5A, stainings of B4GALT1 were undetectable on Day 12 but intensively present in the conceptus trophectoderm and the uterine LE to which the conceptus apposes on Day 15. In addition, FUT4 was weakly detected in the uterine LE on Day 12, but uniform staining of FUT4 was observed in the endometrium and conceptus on Day 15 (Figure 5A). Thus, only the B4GALT1 showed the same spatio-temporal expression patterns as the Le^x^ antigen. Consistently, the qRT-PCR results confirmed that the *B4GALT1* gene exhibited significantly higher expression levels on Day 15 compared to Day 12 in pig endometrium-conceptus tissue samples (Figure 5B). Therefore, the regulatory mechanisms involved in *B4GALT1* gene expression in pig uterine LE-conceptus interface were further investigated. The publicly assessable ChIP-Seq data generated from several pig tissues were reanalyzed [26]. Two H3K27ac-enriched regions and one H3K4me3-enriched region were detected at the 5’ end of the *B4GALT1* gene body, and the sequences of one H3K27ac-enriched region and the H3K4me3-enriched region are conserved among the eight tissues (Figure 5C). Subsequent ChIP-qPCR confirmed that the enrichment of H3K27ac and H3K4me3 in the two regions was significantly increased in pig endometrium-conceptus tissue samples from Day 12 to Day 15 (Figure 5D).

## Discussion

In this study, we identified for the first time the O-glycans from pig endometrium during the attachment stage using the MALDI-MS profiling. O-glycans biosynthesis starts by adding an N-acetylgalactosamine to serine or threonine residues to form the Tn antigen structure. The Tn antigen is then extended to generate different core structures. The complexity of the core structures is increased by the sequential addition of Gal, GlcNAc, GalNAc, Fuc, Sia, and sulfate [30]. The monosaccharide residues at the non-reducing terminal glycan chains usually include Gal, Fuc, and Sia. By comparing our O-glycan data to those generated from other pig tissues published by other groups [18–20], we found that most O-glycans identified in the endometrium and kidneys contain fucose and sialic acid residues (in the form of Neu5Gc). However, the O-glycans identified in the heart and gastric tissues do not have fucose but consist predominantly of sialic acid residues (Supplementary Table 3). Our data support the idea that O-glycan structures are expressed tissue-biasedly.

In the study, many fucosylated O-glycans were identified. The well-known fucose-containing structures include blood group and Lewis antigens. In addition to being present on red blood cells (RBCs), blood group antigens are also distributed on tissues and other non-RBCs [31]. However, little is known about their expressions in the pig maternal-conceptus interface during implantation. Pigs have only two blood group antigens, A and O(H), with A as the more common one [27]. This study revealed that O(H) antigen was weakly but stably present in the glandular epithelium on Days 12 and 15, whereas the A antigen was expressed in pig uterine LE in an inter-individual variation manner, which is similar to the findings in humans that the A antigen expression on tissues varies from individual to individual [31]. One possible cause for the inter-individual variation might be the difference in maternal immunogenic response, which is worth further investigation.

Although Le^x^ and Le^y^ antigens are the two Lewis antigens detected to be expressed in the uterine LE, only Le^x^ antigen was expressed strongly and spatially restricted to the uterine LE-conceptus interface on day 15 of pregnancy. Therefore the functions of the Le^x^ antigen were investigated further. First, it was not expressed in the endometrium on estrous cycle Days 12 and 15, indicating that the ovarian hormone may not be able to induce the expression of Le^x^ antigen. Second, it was expressed at a much lower level in the uterine LE on Day 12 when the maternal recognition of pregnancy occurs. However, wherever the conceptus is located in the uterus, Le^x^ antigen was always strongly expressed in the uterine LE-conceptus interface on Day 15, the early attachment stage during the apposition phase of implantation in pigs. The findings imply an involvement of Le^x^-carrying O-glycans in conceptus attachment in pigs.

Le^x^ antigen is also known as CD15 or SSEA-1 (stage-specific embryonic antigen-1). Besides being a marker of embryonic stem cells and mesenchymal stem cells, it also plays a role in facilitating cell-cell adhesions, which could be mediated by selectins [8, 32–35]. Previously, the contributions of selectins in the attachment of conceptuses to the uterine LE have been determined in other species. For example, the SLe^x^/L-selectin system can mediate the adhesion of the embryo to the uterine LE in humans [11, 36]. However, our study showed that all three members of the selectin family were not expressed in pig uterine LE or conceptus, suggesting that, unlike humans, the Le^x^-carrying O-glycans /selectin system might not participate in the conceptus attachment in pigs.

SPP1 is an adhesion molecule that can bind to multiple integrins. The interactions among SPP1, uterine LE integrin αVβ3, and trophectoderm integrin αVβ6 have been implicated in pig conceptus attachment [2, 37–39]. Like Le^x^ antigen, SPP1 is expressed in the uterine LE-conceptus interface on Day 15, wherever the conceptus is located in the uterus [4]. Our finding that Le^x^ antigen was co-localized with SPP1 in the uterine LE-conceptus interface on Day 15 of pregnancy but not on estrous cycle Day 15 suggests a possibility that Le^x^-carrying O-glycans and SPP1 may work together to affect the conceptus attachment. SPP1 is an extensively post-translationally modified extracellular protein. In addition to containing many phosphorylation sites, SPP1 also consists of several N- and O-glycosylation sites in the threonine/proline-rich region, which are well conserved among mammalian species [40]. Additionally, the O-glycosylation sites proximal to the Arg-Gly-Asp (RGD) region have an impact on the adhesive functions of SPP1 by influencing the association of SPP1 with integrins [40]. Meanwhile, the role of integrins is also highly dependent on their glycosylation status [18, 41, 42]. For example, Le^y^ antigen-carrying integrin αVβ3 has been implicated in human trophoblast-uterine epithelial cell adhesion through activation of integrin αVβ3/FAK signaling [43]. However, the O-glycan structures on SPP1 and integrins have not yet been reported. This study detected an interaction between Le^x^-carrying O-glycans and SPP1, integrin αV, and integrin β6. Due to the fact that terminal fucosylation has many functions in regulating cell-cell communication [44, 45], we could hypothesize a role of Le^x^-carrying O-glycans in conceptus attachment through modulating the SPP1-integrin receptors signaling. Thus, our findings extend the understanding of the regulating mechanisms for SPP1-integrins mediated conceptus attachment, which will be the subject of future research.

We next examined the regulators for the unique spatio-temporal expression pattern of Le^x^ antigen. FUT4 is a fucosyltransferase catalyzing the transfer of α1,3-fucose to Galβ1-4GlcNAcβ1-R for forming Le^x^ antigen (Galβ1-4(Fucα1-3)GlcNAcβ1-) [46, 47]. Although FUT4 showed the same temporal expression pattern with Le^x^ antigen and was expressed in the uterine LE-conceptus interface, its spatial expression pattern was not consistent with Le^x^ antigen because it was also observed in the uterine LE to which the conceptus does not attach. It should be noted that, in addition to Le^x^ antigen, FUT4 also participates in synthesizing other Lewis antigens, such as Le^y^ [44, 45, 48]. Indeed, FUT4 showed a consistent expression pattern with Le^y^ antigen in pig uterine LE. Thus, the discrepancy in spatial expression pattern between FUT4 and Le^x^ antigen could be explained by the possibility that FUT4 may participate in synthesizing both Le^x^ and Le^y^ antigens in pig uterine LE-conceptus interface. In addition, B4GALT1 is a member of the beta-1,4-galactosyltransferase gene family that can catalyze the synthesis of the Galβ1-4GlcNAcβ1-R structure [46, 47]. Notably, it showed an identical spatio-temporal expression pattern with Le^x^ antigen, suggesting that B4GALT1 may be a critical glycosyltransferase that determines the unique spatio-temporal expression pattern of Le^x^ antigen in the uterine LE-conceptus interface during the attachment stage. Subsequently, we confirmed that the *B4GALT1* gene was up-regulated on Day 15. On the other hand, recent studies revealed that the 5’ end of the human *B4GALT1* gene contains multiple open chromatin sites rich in epigenetic regulatory elements [49, 50, 51]. H3K27ac and H3K4me3 are the two epigenetic marks associated with transcriptional activation. In this study, the enrichment of H3K27ac and H3K4me3 was identified at the 5’ end and was associated with the up-regulated level of the *B4GALT1* gene on Day 15. The findings could place the *B4GALT1* gene as a candidate regulator of Le^x^ antigen in the uterine LE-conceptus interface during the attachment stage. Further functional characterization of the regulatory regions identified in *B4GALT1* gene will provide helpful information to elucidate the role of the Le^x^-carrying O-glycans in conceptus attachment.

In conclusion, our results support a hypothesis that Le^x^-carrying O-glycans might play a role in conceptus attachment through modulating the SPP1-integrin receptors signaling. In addition, we identified a candidate regulator that may determine the expression pattern of Le^x^ antigen in pig uterine LE-conceptus interface during the attachment stage. Our findings provide insight into the mechanisms underlying the implantation in pigs.

## Supplementary Material

Supplementary Figure

Supplementary Table

## Figures and Tables

**Table 1:** O-glycans compositions in pig endometrium on Day 15 of pregnancy determined by MALDI-MS.

Peak	Observed m/z	Theoretical m/z	Calculated composition	Proposed structure
1	1024.6	1024.5	Hex_1_.HexNAc_3_	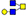
2	1157.7	1157.6	Hex_2_.HexNAc_2_.Fuc_1_	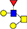
3	1198.8	1198.6	Hex_1_. HexNAc_3_.Fuc_1_	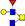
4	1228.8	1228.6	Hex_2_.HexNAc_3_	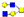
5	1269.8	1269.7	Hex_1_.HexNAc_4_	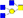
6	1303.8	1303.6	Hex_2_.HexNAc_1_ .Fuc_1_ .Neu5Gc_1_	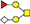
7	1331.8	1331.7	Hex_2_.HexNAc_2_.Fuc_2_	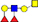
8	1374.8	1374.7	Hex_2_.HexNAc_2_.Neu5Gc_1_	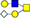
9	1402.9	1402.7	Hex_2_.HexNAc_3_.Fuc_1_	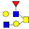
10	1432.9	1432.7	Hex_3_.HexNAc_3_	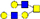
11	1473.9	1473.7	Hex_2_.HexNAc_4_	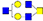
12	1577.0	1576.8	Hex_2_.HexNAc_3_.Fuc_2_	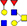
13	1648.0	1647.8	Hex_2_.HexNAc_4_.Fuc_1_	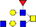
14	1781.1	1780.9	Hex_3_.HexNAc_3_ .Fuc_2_	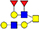
15	1906.2	1906.0	Hex_1_.HexNAc_5_.NeuGc_1_	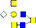
16	2110.4	2110.1	Hex_2_.HexNAc_5_.NeuGc_1_	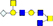
17	2314.4	2314.2	Hex_3_.HexNAc_5_.NeuGc_1_	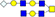

## Data Availability

All data are available in the main text or the supplementary materials.
