# Supplementary material for: Lewis x-Carrying O-glycans are Candidate Modulators for Conceptus Attachment in Pigs
Source: Biol Reprod. Author manuscript; Available in PMC 2023 Feb 17. (PMC7614189; doi:10.1093/biolre/ioac204)
Supplement: Supplementary Figure [file EMS157718-supplement-Supplementary_Figure.pdf]

Supplementary Figures

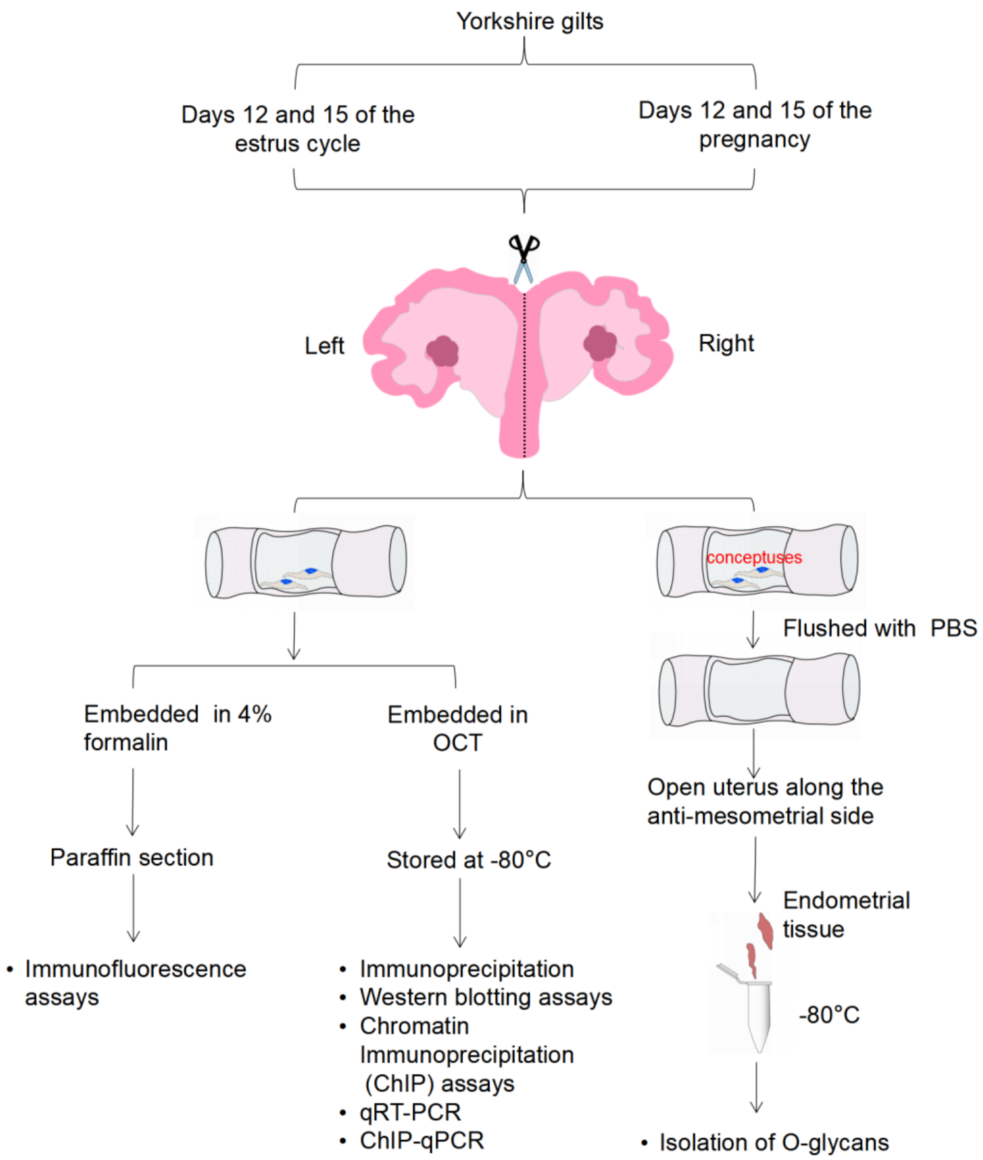

Supplementary Figure 1. A brief schematic diagram of sample collection performed in the study.

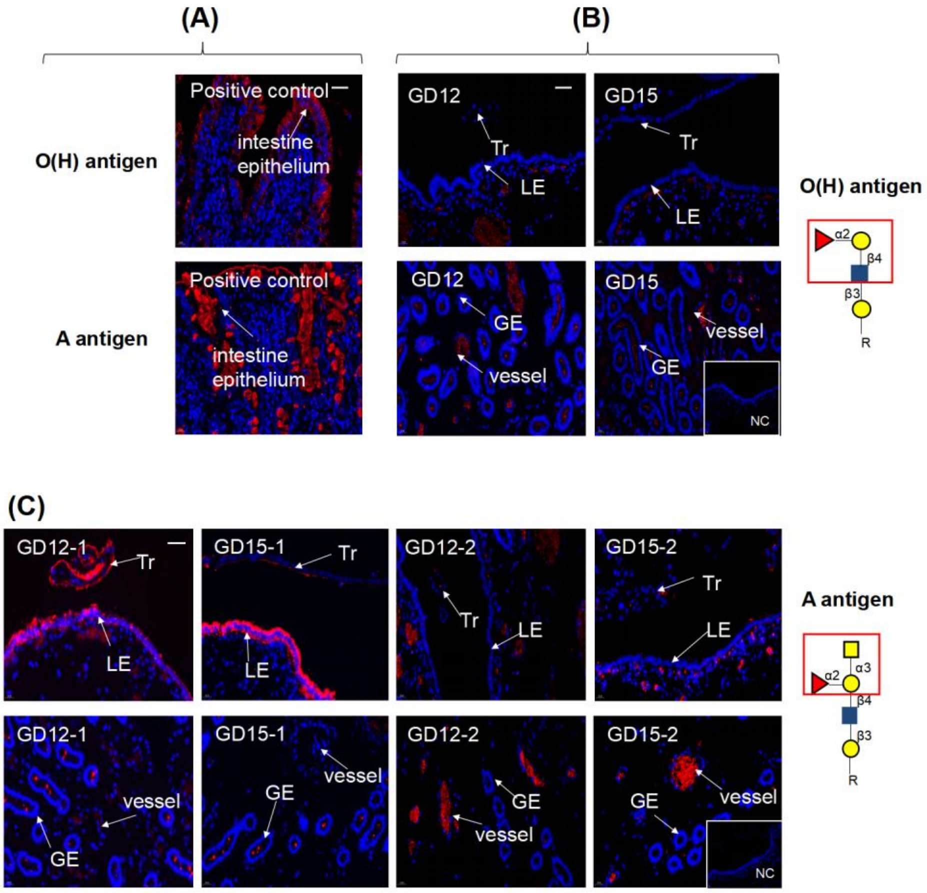

Supplementary Figure 2. The expression of the blood group antigens in pig tissues. (A) Pig intestinal tissues were used as the positive controls. (B) Representative images show the expression of the blood group O(H) antigen in uterine LE and conceptus (top row of the panel) and other compartments of the endometrium (bottom row of the panel) in the uterine cross-sections from Days 12 and 15 of pregnancy in pigs (n = 3 gilts/Day of pregnancy). NC, negative control. (C) Representative images show the expression of the A antigen in uterine LE and conceptus (top row of the panel) and other compartments of the endometrium (bottom row of the panel) in the uterine cross-section from Days 12 and 15 of pregnancy in pigs (n = 4 gilts/Day of pregnancy). NC, negative control. Representative images labeled with GD12-1 and GD15-1 show that uterine LE and glandular LE were positive for blood group A antigen. In contrast, those marked with GD12-2 and GD15-2 show that uterine LE and glandular LE were negative for blood group A antigen. The schematic diagram showing the structure of each antigen detected in this study is next to the corresponding panel. Fucose ▲. N-acetylglucosamine ■. N-acetylgalactosamine ■. Galactose ●. LE, luminal epithelium. GE, glandular luminal epithelium. Tr, trophoblast. GD, Day of pregnancy. Scale bar = 50 µm. The antibodies and the negative controls used are listed in Supplementary Table 1.

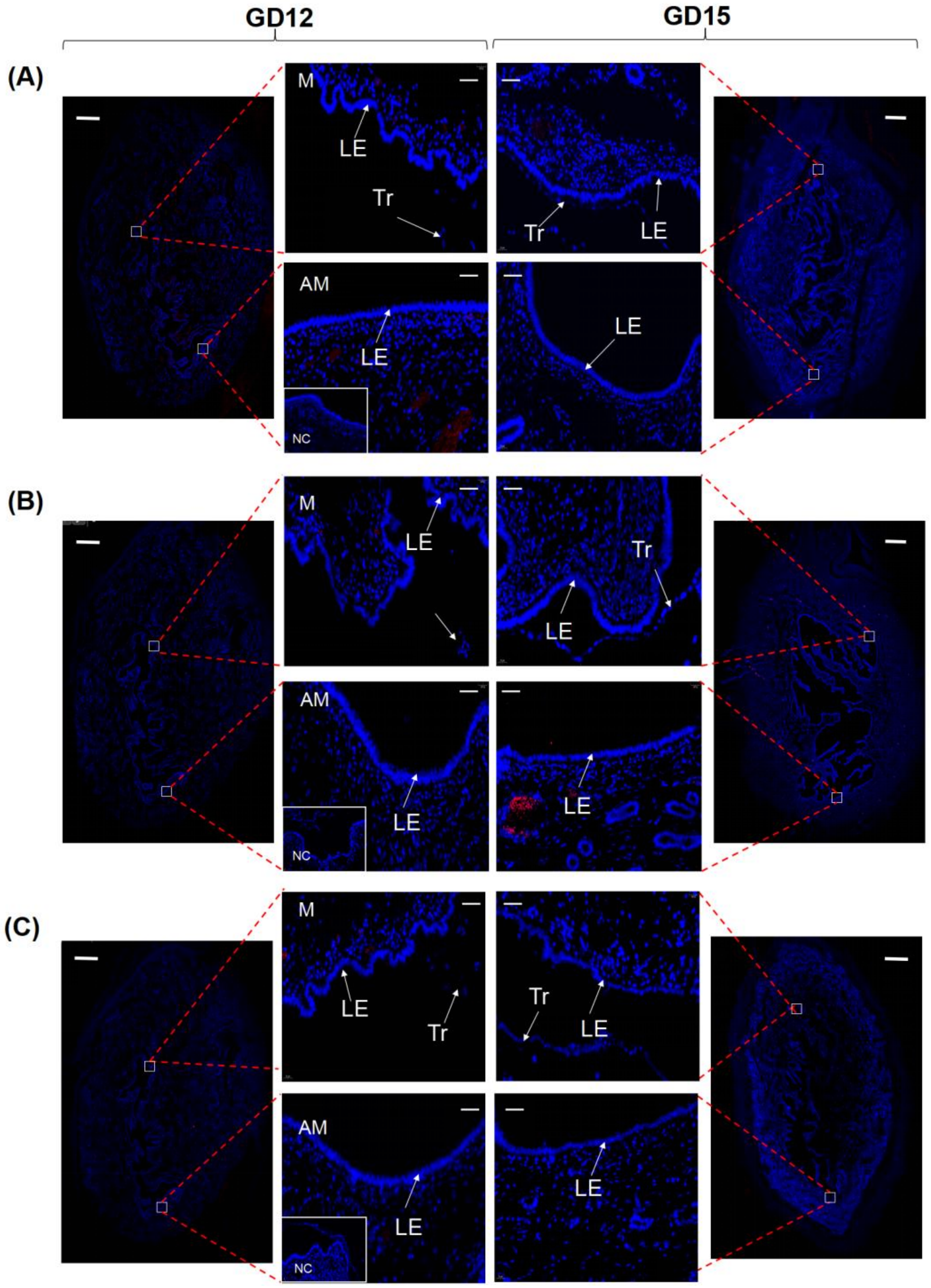

Supplementary Figure 3. Expression of P-selectin (A), E-selectin (B), and L-selectin (C) in the uterine cross-sections from Days 12 and 15 of pregnancy. White squares in each stained uterine cross-section (scale bar = 2000 µm) specify the areas shown at higher magnification (scale bar = 50 µm). NC, negative control. M, mesometrial side. AM, anti-mesometrial side. LE, luminal epithelium. Tr, trophoblast. The antibodies and the negative controls used are listed in Supplementary Table 1.

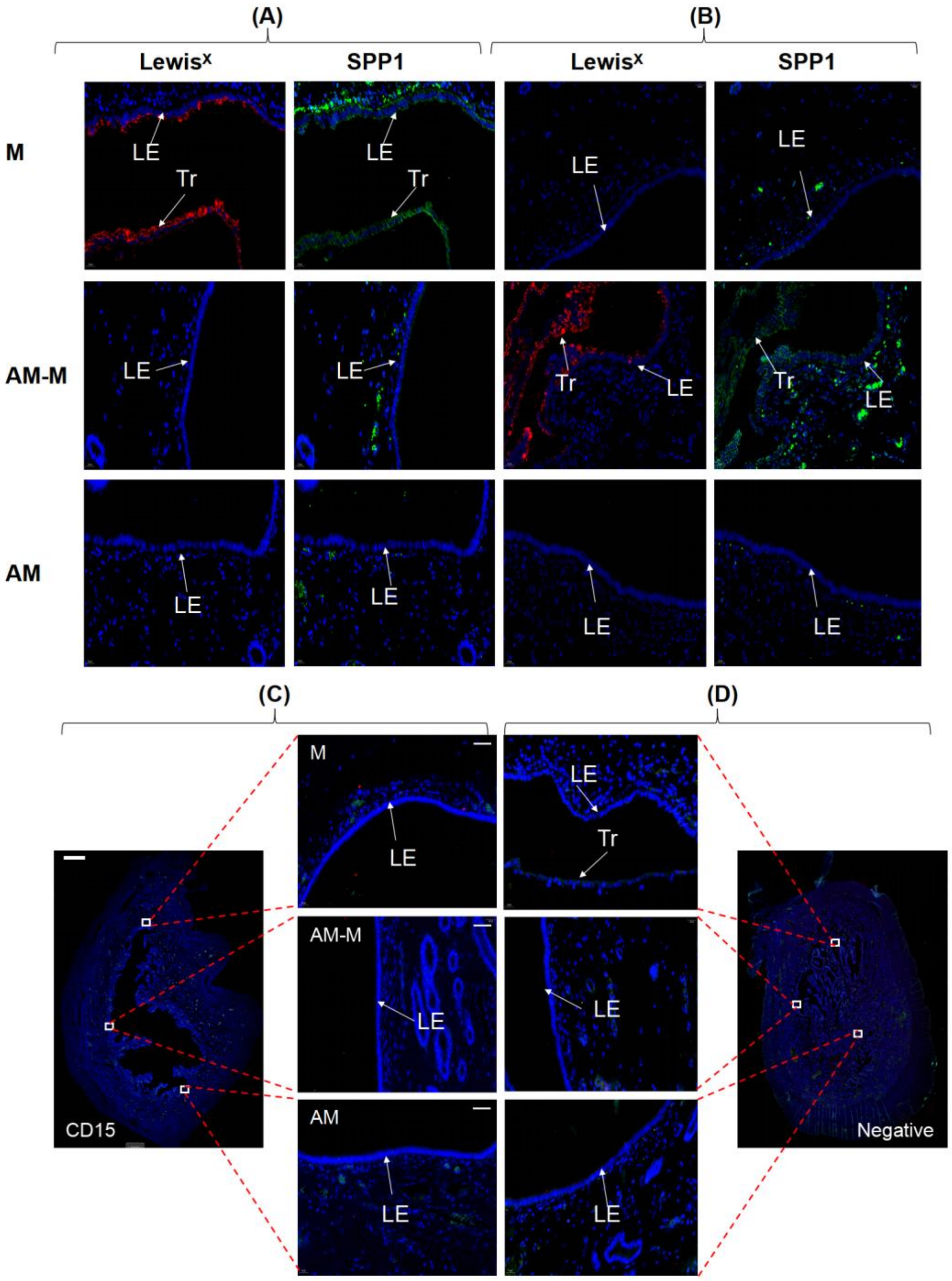

Supplementary Figure 4. Representative images show the individual signal of Le^x-carrying O-glycans (red) and SPP1 (green) separately in the uterine LE-conceptus interface on Day 15 in which the conceptus attaches to the mesometrial side (A) or a site away from the mesometrial side (B). (C) Representative overlay images of the co-localization of Le^x-carrying O-glycans and SPP1 in the uterine cross-sections from estrous cycle Day 15. (D) Representative images show the negative control, which was detected in the uterine cross-sections on Day 15 of pregnancy. M, mesometrial side. AM, anti-mesometrial side. LE, luminal epithelium. Tr, trophoblast. The antibodies and the negative controls used are listed in Supplementary Table 1.

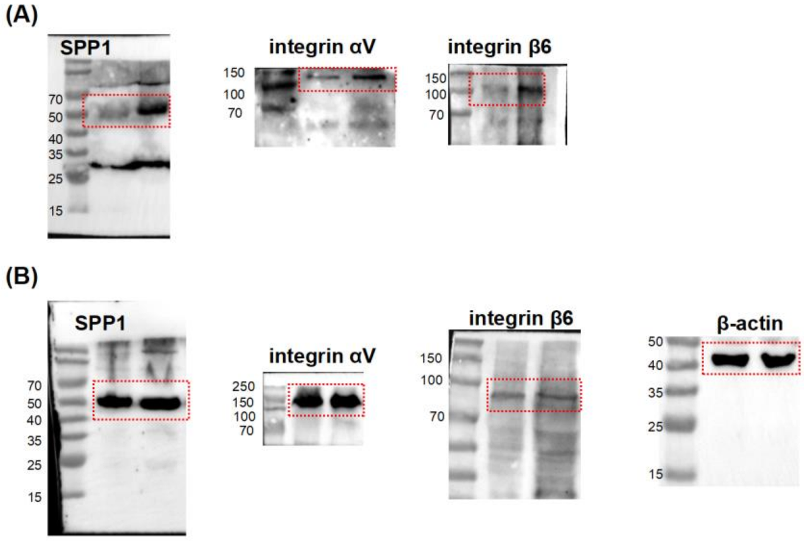

Supplementary Figure 5. The original images of intact gels of Figures 4 C and 4D. The red dotted line indicates the bands shown in Figures 4 C and 4D.

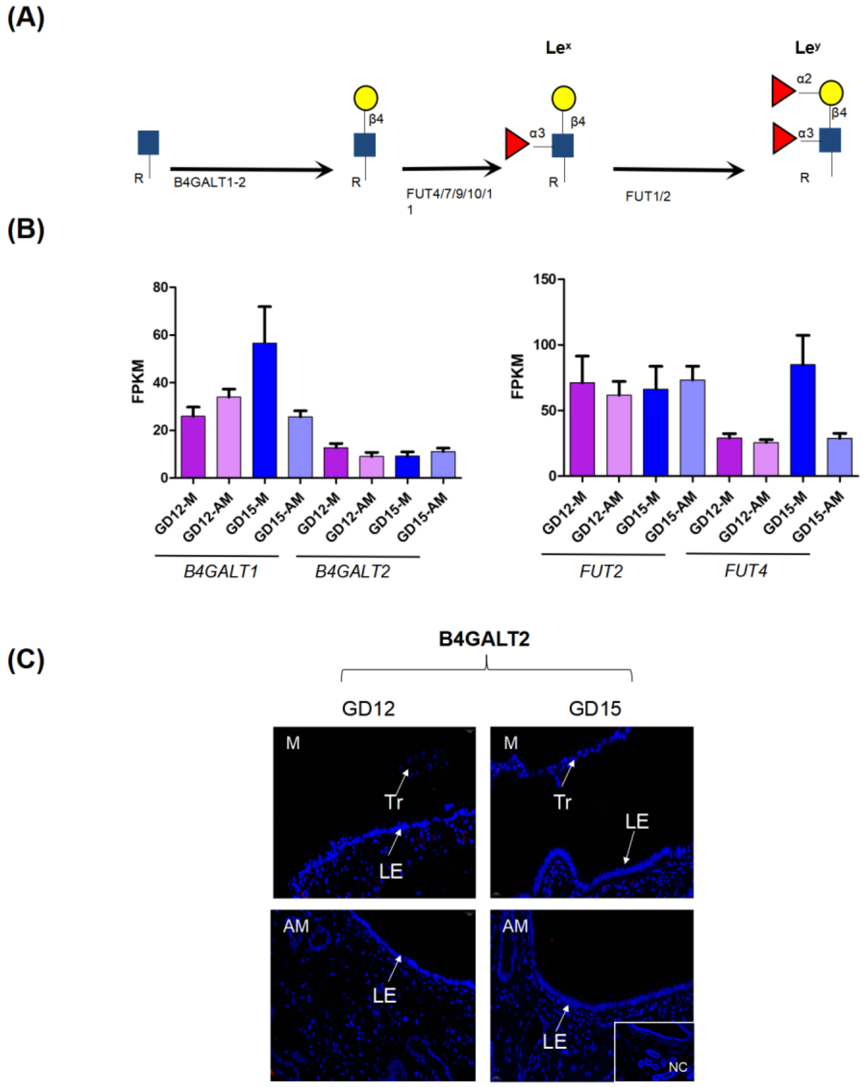

Supplementary Figure 6. Analysis of glycosyltransferase genes involved in the synthesis of Lewis x-carrying O-glycans. (A) The diagram shows the key glycosyltransferases for the biosynthesis of the Lewis x antigens (Data from the Complex Carbohydrate Research Center database). (B) The *B4GALT* and *FUT* genes with FPKM greater than 10 were retrieved from the RNA-seq data [2]. (C) Representative images show the expression of B4GALT2 at the mesometrial side (M; the top row of the panel) and anti-mesometrial side (AM; bottom row of the panel) in the uterine cross-sections from Days 12 and 15 of pregnancy (n = 3 gilts/Day of pregnancy). NC, negative control. M, mesometrial side. AM, anti-mesometrial side. LE, luminal epithelium. Tr, trophoblast. The antibody and the negative controls used are listed in Supplementary Table 1.
