# Supplementary material for: Lewis x-Carrying O-glycans are Candidate Modulators for Conceptus Attachment in Pigs
Source: Biol Reprod. Author manuscript; Available in PMC 2023 Feb 17. (PMC7614189; doi:10.1093/biolre/ioac204)
Supplement: Supplementary Table [file EMS157718-supplement-Supplementary_Table.pdf]

Supplementary Tables

Supplemental Table 1. Information on the antibodies and the negative controls

Primary Antibody	Dilution	Epitope for Carbohydrate-specific antibodies	Source	Catalogue	Nonspecific immunoglobulin used for negative control (Catalogue)	Secondary antibodies used for immunofluorescent staining (Dilution/Catalogue)
Carbohydrate-specific antibodies						
anti-CD15	1:100	Le^x antigen	Abcam, Cambridge, UK	ab665	IgM (ab91117)	Goat anti-Mouse IgM (1:250/A-21426)
anti-Le^y	1:50	Le^y antigen	Abcam, Cambridge, UK	ab3359	IgM (ab91117)	Goat anti-Mouse IgM (1:250/A-21426)
anti-Le^a	1:50	Le^a antigen	Abcam, Cambridge, UK	ab3967	IgM (ab91117)	Goat anti-Mouse IgM (1:250/A-21426)
anti-Le^b	1:50	Le^b antigen	Abcam, Cambridge, UK	ab3968	IgM (ab91117)	Goat anti-Mouse IgM (1:250/A-21426)
anti-H (O)	1:50	blood group H antigen	Invitrogen, Waltham, MA, USA	14-9810-80	IgM (ab91117)	Goat anti-Mouse IgM (1:250/A-21426)
anti-A	1:50	blood group A antigen	Abcam, Cambridge, UK	ab2521	IgM (ab91117)	Goat anti-Mouse IgM (1:250/A-21426)
MECA-79	1:50	6-O-sulfated GlcNAc antigen	Santa Cruz, Heidelberg, Germany	sc-19602	IgM (ab91117)	Goat anti-Mouse IgM (1:250/A-21426)
HECA-479	1:50	SLe^x antigen	Santa Cruz, Heidelberg, Germany	sc-53514	IgM (ab91117)	Goat anti-Mouse IgM (1:250/A-21426)
Protein-specific antibodies						
anti-L-selectin	1:50		Invitrogen, MA, USA	14-0629-82	IgG (A7016)	Goat anti-Rabbit IgG(1:250/A-21429)
anti-E-selectin	1:50		Abcam, Cambridge, UK	ab300557	IgG (A7016)	Goat anti-Rabbit IgG(1:250/A-21429)
anti-P-selectin	1:50		Abcam, Cambridge, UK	ab54427	IgG (A7016)	Goat anti-Rabbit IgG(1:250/A-21429)
anti-B4GALT1	1:50		GeneTex, Irvine, CA, USA	GTX131598	IgG (A7016)	Goat anti-Rabbit IgG(1:250/A-21429)
anti-B4GALT2	1:50		ABclonal Technology, Wuhan, China	A17573	IgG (A7016)	Goat anti-Rabbit IgG(1:250/A-21429)
anti-FUT4	1:50		Proteintech, Wuhan, China	19497-1-AP	IgG (A7016)	Goat anti-Rabbit IgG(1:250/A-21429)
anti-SPP1	1:50/ 1:2500		Abcam, Cambridge, UK	ab214050	IgG (A7016)	Goat anti-Rabbit IgG(1:250/A-21429)
anti-integrin αV	1:2500		Abcam, Cambridge, UK	ab179475		
anti-integrin β6	1:2500		Proteintech, Wuhan, China	19695-1-AP		
anti-integrin β3	1:2500		Abcam, Cambridge, UK	ab179473		
anti-H3K4me3	10ug		Abcam, Cambridge, UK	ab8580		
anti-H3K27ac	10ug		Abcam, Cambridge, UK	ab4729		
anti-β-actin	1:2500		Beyotime, Shanghai, China	AF5003		

Supplementary Table 2: List of primers used for qRT-PCR and ChIP-qPCR.

Name	Primer Sequence (5'->3')	Annealing temperature (℃)	GeneBank Accession No./ Genomic Location (Sscrofa11.1)
B4GALT1	F: TCACTACACCCCCAAGGACT<br>R:GCACGGTTGAACATGGATTCT	55.6	XM_003130680.4
B4GALT1-H2K27ac	F: CTGCTCGGTAAGGATTCGGG<br>R: CAGCCTCTAACACCAGTCCG	59.5	Chr10:33,317,601-33,318,122
B4GALT1-H2K4me3	F: CCGCCAGCAACTTGACTT<br>R: CCTCTGTTTGGGATTAGGGACTC	59.5	Chr10:33,317,430-33,318,078

Supplemental Table 3. List the number of fucose-containing O-glycans identified in different porcine tissues.

Tissue	Total number[1]	Mono-fucose-containing	Di-fucoses-containing	Tri-fucoses-containing	Sialic acid-containing	Reference
Pericardium	20	0	0	0	17	[18]
Pulmonary	31	0	0	0	20	[18]
Aortic	23	0	0	0	15	[18]
Gastric	8	0	0	0	1	[19]
Kidney	28	1	10	3	13	[20]
Endometrium	18	7	5	0	2	This study

[1]Total number of the O-glycan compositions identified
